# Global Trends in Antimicrobial Resistance-Related Mortality from *Klebsiella pneumoniae*-Associated Lower Respiratory Infections: A GBD 2021 Analysis, 1990–2021

**DOI:** 10.3390/tropicalmed11080222

**Published:** 2026-08-10

**Authors:** Hongquan Chen, Shiyi Wang, Min Yuan, Zhiguo Liu, Zhenjun Li

**Affiliations:** National Key Laboratory of Intelligent Tracking and Forecasting for Infectious Diseases, National Institute for Communicable Disease Control and Prevention, Chinese Center for Disease Control and Prevention & Chinese Academy of Preventive Medicine, Beijing 102200, China; 13843202849@163.com (H.C.); wangshiyi621@126.com (S.W.); yuanmin@icdc.cn (M.Y.); liuzhiguo@icdc.com (Z.L.)

**Keywords:** *Klebsiella pneumoniae*, lower respiratory infections, antimicrobial resistance, global burden of disease, age-standardized mortality rates

## Abstract

*Klebsiella pneumoniae* (*K. pneumoniae*) is a leading cause of hospital- and community-acquired lower respiratory infections (LRI). The emergence of antimicrobial resistance (AMR), especially carbapenem-resistant strains (CRKP), poses a serious global health threat. Using Global Burden of Disease (GBD) 2021 data, we estimated AMR-related mortality for *K. pneumoniae*-associated LRI across regions from 1990 to 2021. Joinpoint regression analysis identified temporal trends. Global mortality declined overall, with a 70% reduction in under-5 deaths but a doubling among adults over 70 years. Deaths associated with and attributable to AMR decreased by 18.9% and 4.5%, respectively. The age-standardized mortality rate (ASMR) fell by nearly 50%. In contrast, the mortality burden associated with carbapenem resistance increased markedly, with AMR-associated deaths rising by 157% and disproportionately affecting high-burden regions such as Sub-Saharan Africa and South Asia. Despite overall progress, the increasing mortality burden associated with carbapenem resistance in *K. pneumoniae*-associated LRI poses a critical and persistent global health threat.

## 1. Introduction

*Klebsiella pneumoniae* (*K. pneumoniae*), an important Gram-negative opportunistic pathogen, commonly colonizes the human gut with rates ranging from 5% to 38% [1]. In immunocompromised hosts, *K. pneumoniae* may cause severe infections such as pneumonia, urinary tract infections, and bloodstream infections [2]. *K. pneumoniae* is an important bacterial cause of lower respiratory infections (LRI), including hospital-acquired pneumonia [3]. Clinical studies demonstrate that LRI carry high mortality rates, particularly among intensive care unit (ICU) patients and elderly individuals [4]. *K. pneumoniae* pneumonia is associated with substantial mortality in critically ill patients. In a retrospective cohort of 285 patients with *K. pneumoniae* ICU-acquired pneumonia, 50 patients died within 30 days [5].

In recent decades, inappropriate antibiotic use has accelerated antimicrobial resistance (AMR) in *K. pneumoniae*, posing a critical global health crisis [6]. In 2019, *K. pneumoniae* infections across multiple infectious syndromes were estimated to account for approximately 800,000 deaths globally, of which approximately 80% were associated with antimicrobial resistance. Lower respiratory infections accounted for approximately 276,000 deaths, representing 34.5% of the total mortality burden due to *K. pneumoniae* infections [7]. Surveillance across 17 countries reveals alarming resistance rates among hypervirulent *K. pneumoniae* strains to third-generation cephalosporins (cefotaxime 65.8%; ceftazidime 57.1%) and carbapenems (imipenem 45.7%; meropenem 51.0%; ertapenem 40.6%) [8]. The spread of carbapenem-resistant *K. pneumoniae* (CRKP) [9], particularly the hypervirulent ST11 clone in China [10,11], and the emergence of multidrug-resistant (MDR) and extensively drug-resistant (XDR) strains have led to near-pan-resistance, significantly increasing mortality, disease severity, and healthcare burdens [12,13].

Previous GBD studies have quantified the global burden and aetiological distribution of lower respiratory infections, including the burden attributable to *K. pneumoniae*. However, age-, region-, and antibiotic-class-specific temporal patterns of the mortality burden associated with and attributable to antimicrobial-resistant *K. pneumoniae* lower respiratory infections remain incompletely characterized [14]. Most available data derive from localized studies or short-term surveillance, failing to provide a global perspective. To address these gaps, this study utilized data from the GBD 2021 study to systematically evaluate AMR burden in *K. pneumoniae*-associated LRI. Through multidimensional analyses encompassing global, regional, age-, sex-, and antibiotic-class-specific patterns, we provide a comprehensive characterization of the epidemiology and spatiotemporal dynamics of AMR in this important pathogen.

## 2. Materials and Methods

### 2.1. Data Sources and Case Definitions

Data on AMR in *K. pneumoniae* were sourced from the GBD 2021 study. The original dataset, compiled from multiple surveillance systems across 204 countries and territories, provides comprehensive estimates of deaths and age-standardized mortality rates (ASMR) associated with and attributable to bacterial AMR for 11 infectious syndromes, 16 antimicrobial classes, and 84 pathogen–drug combinations [15]. Following the GBD 2021 AMR framework, these two AMR burden measures were defined using separate counterfactual scenarios. The burden attributable to AMR was estimated under a counterfactual scenario in which all drug-resistant infections were replaced by drug-susceptible infections, whereas the burden associated with AMR was estimated under a counterfactual scenario in which all drug-resistant infections were replaced by no infection. Thus, the attributable burden represents the excess burden specifically due to resistance, whereas the associated burden represents the broader burden related to drug-resistant infections. The GBD AMR framework systematically quantified cause-specific fatal burdens for various infectious syndromes, including LRI. LRI were defined as acute pneumonia or bronchiolitis, excluding COVID-19-related infections [3]. The extracted indicators included annual death counts, age-standardized mortality rates (ASMR), percentage changes, and estimates stratified by region, age, sex, and antibiotic class. All GBD estimates were obtained directly from the GBD 2021 Results Tool and are reported with their corresponding 95% confidence intervals (CI) to reflect modelling uncertainty. The ASMR was expressed as deaths per 100,000 population.

Six antibiotic classes available for *K. pneumoniae* in the GBD 2021 AMR dataset were included: aminoglycosides, β-lactam/β-lactamase inhibitors, carbapenems, fluoroquinolones, third-generation cephalosporins, and trimethoprim–sulfamethoxazole. Antibiotic-class-specific burden estimates were not mutually exclusive because a single infection could involve resistance to multiple antibiotic classes. Consequently, deaths estimated for individual antibiotic classes may overlap and should not be summed to derive the overall AMR burden.

### 2.2. Average Annual Percentage Change (AAPC)

Temporal trends from 1990 to 2021 were analysed using the Joinpoint Regression Program (version 5.1.0.0; National Cancer Institute, Bethesda, MD, USA). A log-linear model with an uncorrelated error structure was fitted separately for males, females, and both sexes combined. The number of joinpoints was allowed to range from zero to seven, and the optimal model was selected using the weighted Bayesian information criterion. Annual percentage changes (APCs) with 95% CI were estimated for each segment, and average annual percentage changes (AAPCs) were calculated for the entire study period. Trends were considered statistically significant when the 95% CI excluded zero.

### 2.3. Statistical Analysis

All analyses were conducted in R software (version 4.3.1), including data cleaning, computation, and visualization. Figures were generated using the ggplot2 package and finalized with Adobe Illustrator (version CS5).

## 3. Results

### 3.1. Temporal Trends in Non-AMR-Specific Estimates of Global K. pneumoniae-Related LRI Mortality, 1990–2021

**Figure 1:**
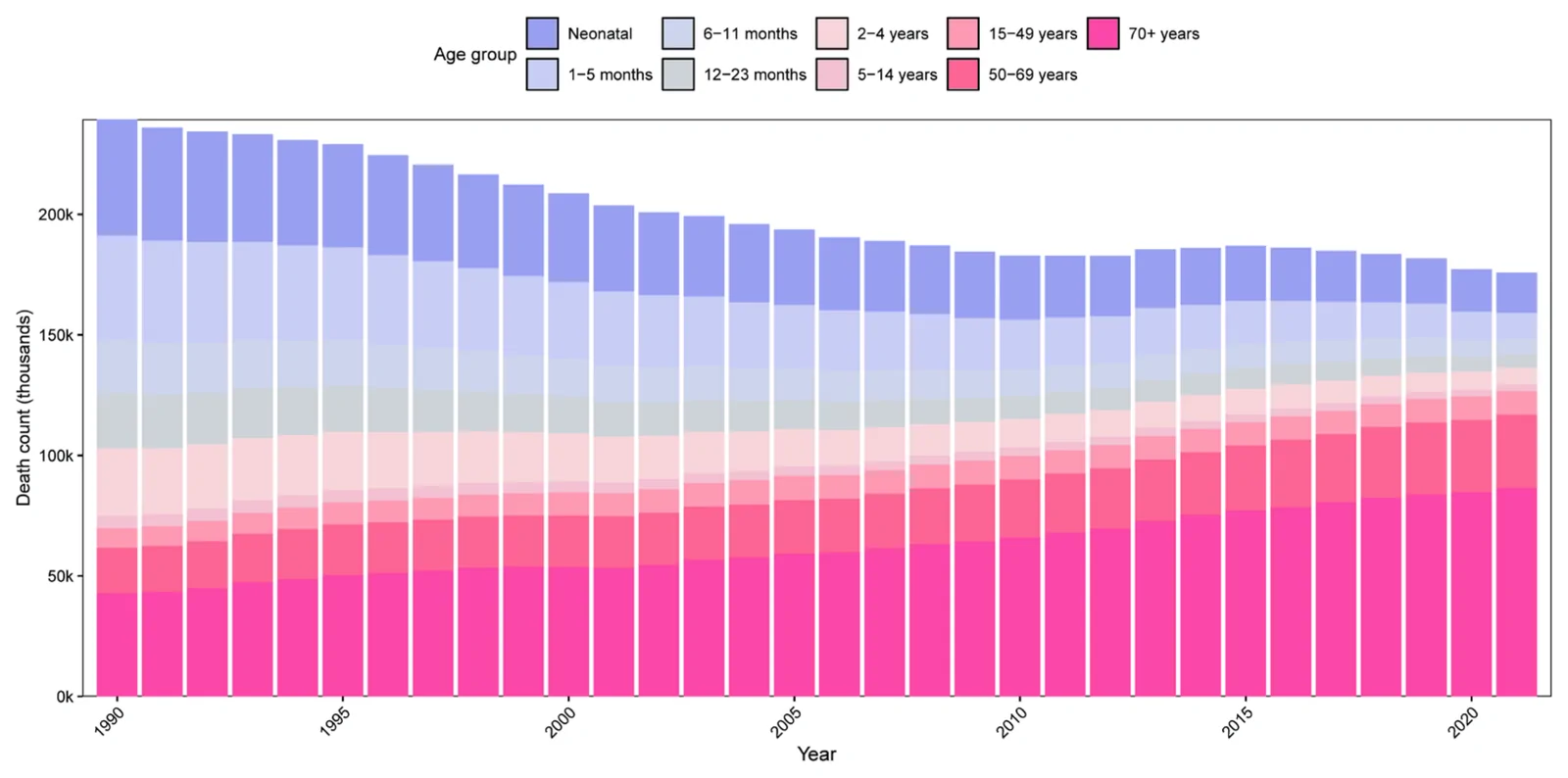
Global time trend of *Klebsiella pneumoniae*-associated lower respiratory infections, by age, 1990–2021. Bar labels represent the number of deaths from *Klebsiella pneumoniae*-associated lower respiratory infections for each year, stratified by the following age groups: neonates, 1–5 months, 6–11 months, 12–23 months, 2–4 years, 5–14 years, 15–49 years, 50–69 years, and over 70 years.

In the non-AMR-specific GBD estimates, the global mortality burden of *K. pneumoniae*-related LRI declined between 1990 and 2021 (Figure 1). In 2021, there were 175,783 deaths (95% CI: 158,749–193,924), representing a 26.6% decrease (95% CI: 16.9–34.1%) from the 239,367 deaths (95% CI: 212,553–268,072) recorded in 1990. The ASMR fell from 4.79 (95% CI: 4.31–5.27) to 2.32 (95% CI: 2.09–2.58) per 100,000 population, corresponding to a 51.5% reduction (95% CI: 46.1–55.9%) (Table S1). Neonates and infants aged 1–5 months remained the highest-risk groups; however, deaths in these groups declined from 48,117 (95% CI: 37,165–59,197) and 43,338 (95% CI: 37,371–49,501) in 1990 to 16,647 (95% CI: 12,863–21,139) and 10,856 (95% CI: 9113–12,653) in 2021, respectively. In contrast, deaths increased across all adult age groups. Between 1990 and 2021, mortality increased by 23.8% among individuals aged 15–49 years (from 7978 to 9877 deaths), by 60.7% among those aged 50–69 years (from 18,795 to 30,209 deaths), and by 102.0% among adults aged 70 years or older (from 42,853 to 86,570 deaths) (Table S2).

### 3.2. Spatiotemporal Trends in AMR Burden of K. pneumoniae-Associated LRI at Global and Regional Levels

**Figure 2:**
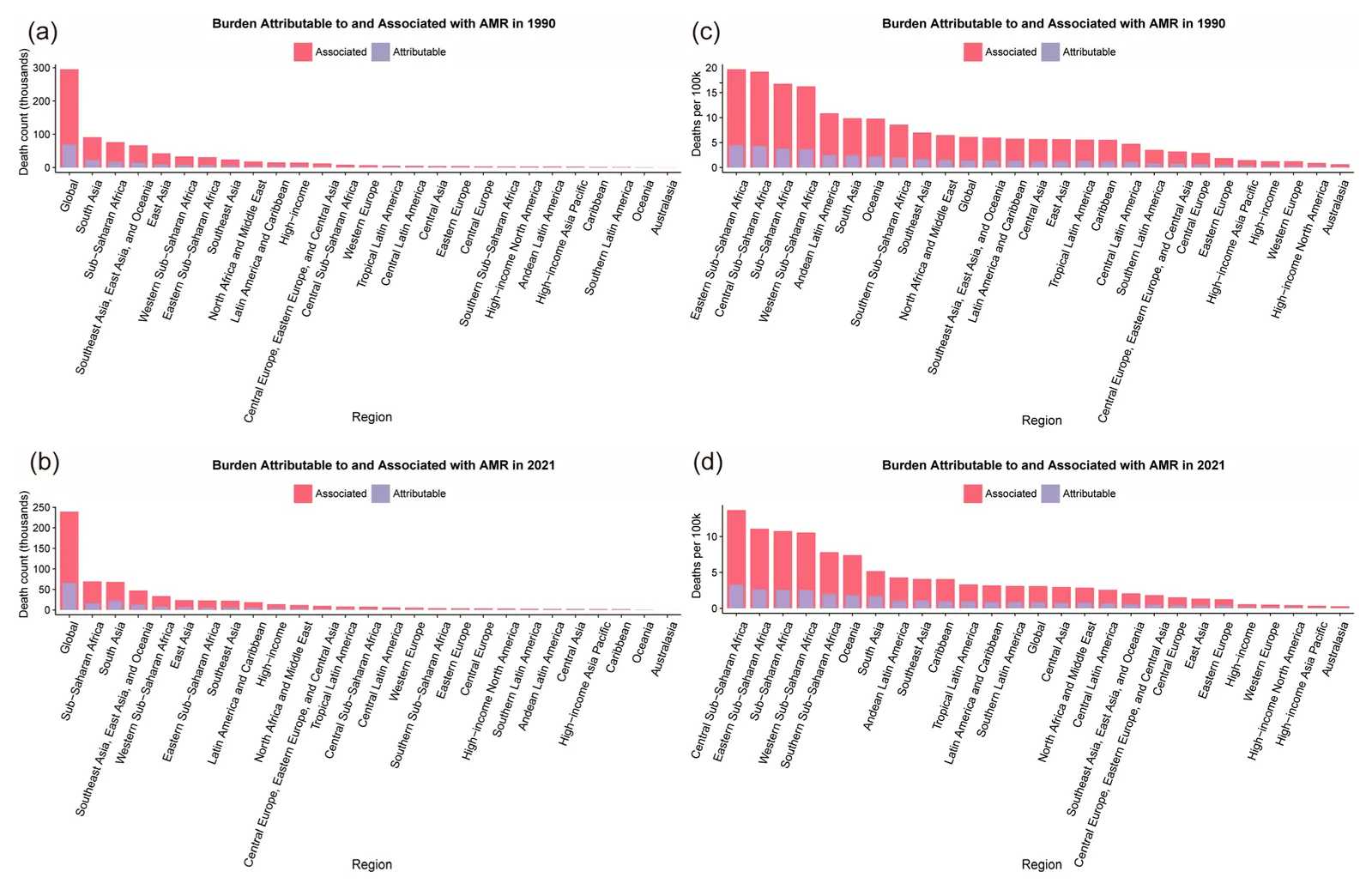
Mortality burden associated with and attributable to bacterial AMR across GBD super-regions and regions, 1990–2021. (**a**,**c**) All-age death counts; (**b**,**d**) Age-standardized mortality rates (ASMR, per 100,000 population).

In the separate GBD AMR framework, the estimated mortality burden associated with and attributable to AMR declined between 1990 and 2021. Under the no-infection counterfactual, AMR-associated deaths decreased by 18.9%, from 295,300 (95% CI: 252,972–337,627) in 1990 to 239,375 (95% CI: 212,831–265,918) in 2021 (Figure 2a,b), while the ASMR fell more substantially by 49.6%, from 6.127 (95% CI: 5.282–6.972) to 3.09 (95% CI: 2.735–3.446) per 100,000 (Table S3, Figure 2c,d). Deaths attributable to AMR declined modestly by 4.5%, from 69,215 to 66,133, though the corresponding ASMR decreased by 40.7%, from 1.435 to 0.851 per 100,000 (Table S3).

In 2021, Sub-Saharan Africa and South Asia recorded the highest number of AMR-associated deaths, at 69,454 (95% CI: 57,497–81,410) and 68,082 (95% CI: 60,356–75,807), respectively. However, their AMR-attributable burdens differed substantially, with 16,593 deaths (95% CI: 13,163–20,023) in Sub-Saharan Africa and 21,929 deaths (95% CI: 18,858–24,999) in South Asia. Accordingly, the ratio of AMR-attributable to AMR-associated deaths was approximately 23.9% in Sub-Saharan Africa, compared with 32.2% in South Asia, indicating marked regional variation in the relative contribution of resistance within the GBD counterfactual framework. At the regional level, Central Sub-Saharan Africa had the highest ASMR for both AMR-associated mortality (13.696 per 100,000; 95% CI: 10.767–16.625) and AMR-attributable mortality (3.281 per 100,000; 95% CI: 2.470–4.092). High ASMRs were also observed in Eastern and Western Sub-Saharan Africa and in Sub-Saharan Africa overall (Table S3).

### 3.3. AMR Burden by Antibiotic Classes in K. pneumoniae-Associated LRI

**Figure 3:**
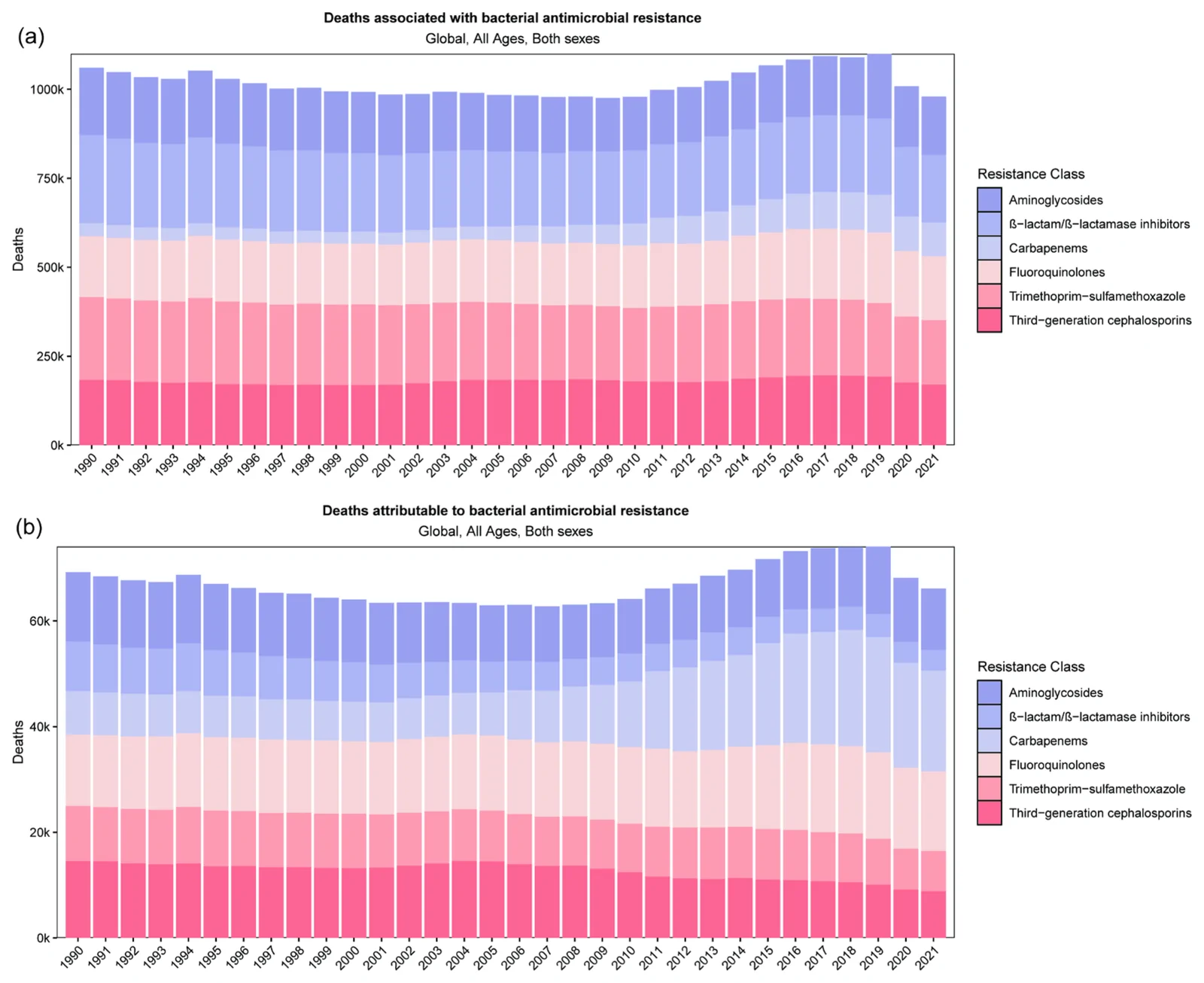
Global temporal trends in all-age deaths from *Klebsiella pneumoniae*-associated lower respiratory infections across six antibiotic classes, 1990 to 2021. Bar labels represent the all-age death count for six antibiotic classes (**a**) associated with and (**b**) attributable to bacterial AMR.

Mortality burdens were estimated separately for each of the six antibiotic classes. These class-specific estimates were non-additive because infections with resistance to multiple antibiotic classes could contribute to more than one class-specific burden estimate. Therefore, values across antibiotic classes should not be summed to derive the overall AMR burden. Globally in 2021, resistance to five antibiotic classes—aminoglycosides, β-lactam/β-lactamase inhibitors, fluoroquinolones, trimethoprim–sulfamethoxazole, and third-generation cephalosporins—was each associated with over 100,000 deaths from *K. pneumoniae*-associated LRI (Table S4). From 1990 to 2021, both deaths associated with AMR and ASMR for these five classes generally declined (Figure 3a and Figure S1a). The largest reductions were observed for β-lactam/β-lactamase inhibitors, with deaths decreasing by 23.2% and ASMR by 51.0%, and for trimethoprim–sulfamethoxazole, with a 22.2% reduction in deaths and a 50.7% reduction in ASMR. In contrast, deaths and ASMR associated with carbapenem resistance increased substantially by 157.1% and 55.9%, respectively.

Among deaths attributable to AMR, carbapenem resistance represented the largest burden in 2021 (Figure 3b), with 19,089 deaths (95% CI: 14,910–23,268)—a 131.3% increase from 1990. The corresponding ASMR increased by 38.9% over this period. Fluoroquinolone resistance was the second-highest contributor, with 15,045 deaths (95% CI: 10,610–19,479) in 2021, marking an 11.4% increase since 1990 despite a 33.0% decline in its ASMR (Figure S1b). The remaining four antibiotic classes all showed consistent decreases in both attributable deaths and ASMRs. The most notable decline was observed for β-lactam/β-lactamase inhibitors resistance, with deaths decreasing by 58.2% (95% CI: 57.0–62.0%) and the ASMR by 72.8% (95% CI: 72.1–76.1%) (Table S4).

### 3.4. Age and Sex Differences in the AMR Burden of K. pneumoniae-Associated LRI

**Figure 4:**
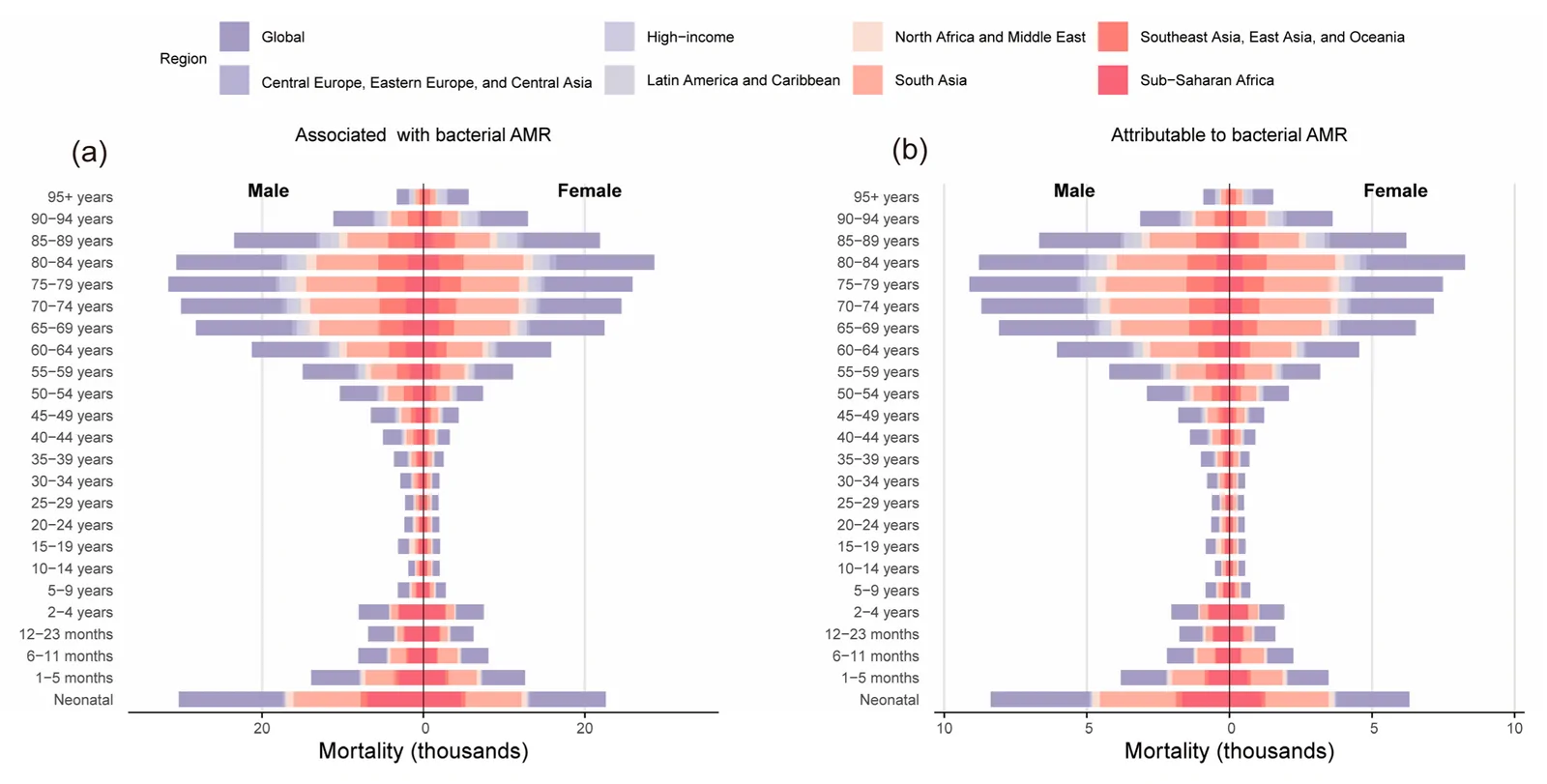
Mortality burden of *Klebsiella pneumoniae*-associated lower respiratory infections in 2021, by age groups, sex, and GBD super-regions. Bar labels represent count of deaths (**a**) associated with and (**b**) attributable to bacterial AMR.

In 2021, the absolute AMR-related mortality burden from *K. pneumoniae*-associated LRI was concentrated at the extremes of age, with the highest numbers of deaths occurring among children younger than 5 years and adults aged 70 years or older (Figure 4a,b). Geographically, Sub-Saharan Africa had the highest number of AMR-associated deaths among children younger than 5 years (30,647; 95% CI: 22,598–38,696), whereas South Asia had the highest number among adults aged 70 years or older (31,045; 95% CI: 26,737–35,353) (Table S5). A similar geographic pattern was observed for AMR-attributable mortality: Sub-Saharan Africa had the highest burden among children younger than 5 years (7314 deaths; 95% CI: 5268–9360), whereas South Asia had the highest burden among adults aged 70 years or older (10,044 deaths; 95% CI: 8438–11,649) (Table S6).

Antibiotic-class-specific patterns differed by age and region. Among children younger than 5 years, resistance to β-lactam/β-lactamase inhibitors and trimethoprim–sulfamethoxazole accounted for the largest numbers of AMR-associated deaths globally, with the highest regional burdens occurring in Sub-Saharan Africa. For AMR-attributable mortality, carbapenem resistance (3439 deaths; 95% CI: 2511–4367) and fluoroquinolone resistance (3062 deaths; 95% CI: 1989–4135) were the leading contributors globally. However, their geographic distributions differed: the largest carbapenem-attributable burden occurred in South Asia, whereas the largest fluoroquinolone-attributable burden occurred in Sub-Saharan Africa. The high aggregate burden among children in Sub-Saharan Africa was distributed across multiple antibiotic classes, with fluoroquinolone and third-generation cephalosporin resistance being the largest contributors. Among adults aged 70 years or older, carbapenem and fluoroquinolone resistance were the leading contributors to AMR-attributable mortality, with the largest regional burdens occurring in South Asia (Tables S7 and S8; Figures S2–S4).

### 3.5. Joinpoint Regression Analysis

**Figure 5:**
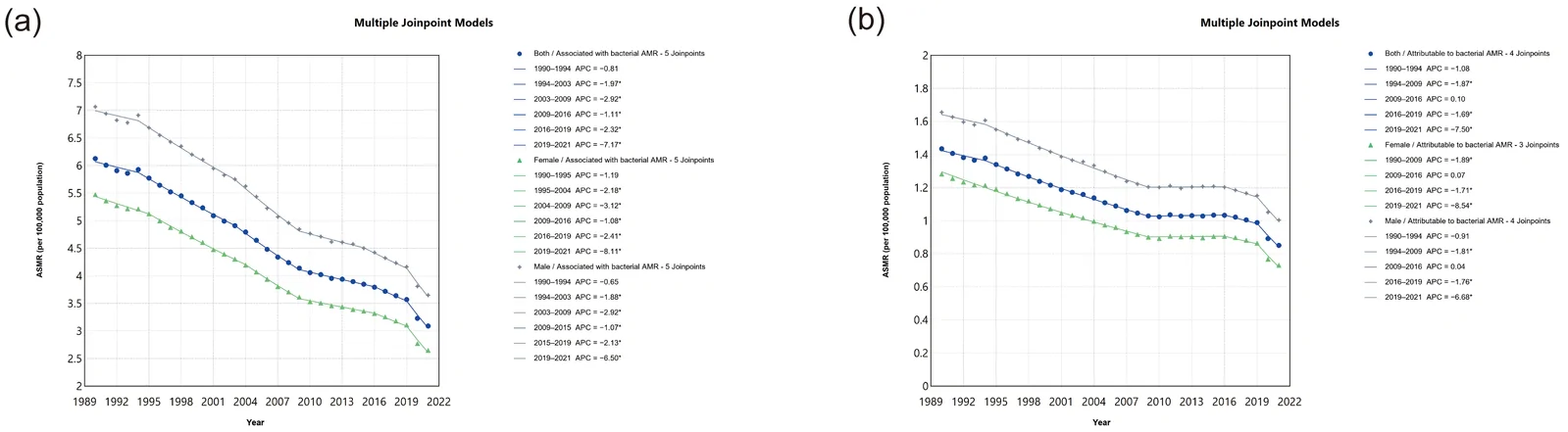
Joinpoint regression analysis of ASMR trends for *Klebsiella pneumoniae*-associated lower respiratory infections with AMR, stratified by sex, 1990–2021. ASMR trends: (**a**) associated with and (**b**) attributable to bacterial AMR. * indicates that the APC is significantly different from zero (*p* < 0.05).

Joinpoint regression analysis revealed a significant overall decline in the ASMR associated with AMR in *K. pneumoniae*-associated LRI from 1990 to 2021 (AAPC = −2.19%; 95% CI: −2.26 to −2.11%; *p* < 0.001). The steepest decrease occurred from 2019 to 2021 (APC = −7.17%; 95% CI: −8.26 to −5.74%; *p* < 0.001) (Figure 5a, Table S9). The ASMR attributable to AMR also exhibited a downward trend (AAPC = −1.68%; 95% CI: −1.76 to −1.60%; *p* < 0.001), with the largest decline from 2019 to 2021 (APC = −7.50%; 95% CI: −8.63 to −6.35%; *p* < 0.001). In contrast, the period from 2009 to 2016 remained relatively stable (APC = 0.10%; 95% CI: −0.11 to 0.79%; *p* = 0.209) (Figure 5b, Table S9).

Analysis by antibiotic class showed that the ASMR related to aminoglycosides, β-lactam/β-lactamase inhibitors, fluoroquinolones, trimethoprim–sulfamethoxazole, and third-generation cephalosporins all decreased overall. In contrast, the ASMR associated with carbapenem resistance increased (AAPC = 1.54%; 95% CI: 1.31–1.76%; *p* < 0.001), characterized by a sharp rise from 2004 to 2012 (APC = 8.33%; 95% CI: 7.40–10.86%; *p* = 0.002) followed by a decline from 2018 to 2021 (APC = −6.61%; 95% CI: −9.75 to −4.16%; *p* < 0.001) (Figure S5, Table S10). A similar fluctuating pattern was observed for the ASMR attributable to carbapenem resistance, which also increased overall (AAPC = 1.07%; 95% CI: 0.94–1.18%; *p* < 0.001), with a pronounced peak around 2009–2012 (APC = 10.62%; 95% CI: 4.43–11.83%; *p* < 0.001) (Figure S6, Table S11). The ASMR was consistently higher in males than in females across all antibiotic classes.

## 4. Discussion

Utilizing data from the GBD 2021 study on AMR, this research systematically analyzed the temporal trends in the global and regional burden of *K. pneumoniae*-associated LRI related to AMR. From 1990 to 2021, the overall global burden of *K. pneumoniae*-associated LRI declined, with substantial reductions observed in both deaths associated with AMR and ASMR. The marked decline in mortality among children younger than 5 years was likely multifactorial, potentially reflecting improvements in neonatal and pediatric care, hygiene and infection prevention, nutrition, access to effective antibiotics, and pneumonia and sepsis management. Although *Haemophilus influenzae type b* (*Hib*) and pneumococcal conjugate vaccines do not directly target K. pneumoniae, these programmes may have indirectly contributed to reductions in severe childhood respiratory infections by reducing coinfections and secondary pneumonia. However, their specific contribution to the observed decline in K. pneumoniae-associated LRI mortality could not be evaluated in the present study [16,17,18]. In contrast, mortality among adults aged 70 years or older more than doubled between 1990 and 2021. Importantly, deaths also increased by 23.8% among adults aged 15–49 years and by 60.7% among those aged 50–69 years. These findings indicate that the upward temporal trend extended across working-age and older adult groups, although the largest absolute burden in 2021 remained among adults aged 70 years or older. This age-related pattern may partly reflect population ageing and the greater susceptibility of older adults to severe *K. pneumoniae*-associated LRI because of comorbidities such as chronic obstructive pulmonary disease and diabetes [19].

Although the overall mortality burden of *K. pneumoniae*-associated LRI has declined, deaths linked to carbapenem resistance increased by 157% over the past three decades, in sharp contrast to the reductions seen for most other antibiotic classes. The ASMR associated with resistance to β-lactam/β-lactamase inhibitors decreased by 51.0%, while the corresponding AMR-attributable ASMR decreased by 72.8% between 1990 and 2021. This decline may be attributed to the development and widespread use of agents such as clavulanic acid, tazobactam, and avibactam [20], as well as newer cephalosporins and quinolones with broader spectra and lower resistance potential [21]. Strengthened antibiotic stewardship and robust AMR surveillance have further curbed misuse and slowed resistance [22]. One possible explanation is that changes in antibiotic-prescribing practices may have increased reliance on carbapenems in some settings, potentially contributing to the selection of carbapenem resistance. However, antibiotic consumption was not evaluated in the present study, and this hypothesis cannot be directly tested using the GBD burden estimates [23]. Carbapenemase genes such as blaKPC and blaNDM can disseminate through mobile genetic elements and high-risk *K. pneumoniae* clones, facilitating regional and international spread [24,25]. Alarmingly, the emergence of hv-CRKP, especially ST11, has driven fatal pneumonia outbreaks in China [10,11], underscoring its escalating global health threat.

From a geographic perspective, the differentiation in the burden of these resistance types further translated into heterogeneity in disease burden at the regional level. In 2021, the AMR-attributable mortality burden from *K. pneumoniae*-associated LRI was particularly high in Sub-Saharan Africa and South Asia. Despite similar numbers of AMR-associated deaths in the two super-regions, the attributable-to-associated mortality ratio was substantially lower in Sub-Saharan Africa than in South Asia (23.9% vs. 32.2%). Within the GBD counterfactual framework, this asymmetry may reflect regional differences in baseline infection severity, pathogen–drug distributions, and access to timely diagnosis and effective treatment. However, these factors were not directly assessed in the present analysis, and the observed difference should not be interpreted as evidence of any specific causal mechanism. The ASMR in Central Sub-Saharan Africa reached 13.696 per 100,000 (95% CI: 10.767–16.625), underscoring the substantial AMR burden in these regions. This regional clustering may partly reflect disparities in healthcare resources. Many low-resource settings in Sub-Saharan Africa face important gaps in access to clinical bacteriology and antimicrobial-susceptibility testing, increasing reliance on modelled estimates and potentially limiting pathogen-directed treatment [26]. Antibiotic consumption has increased substantially in many low- and middle-income countries, although our study did not include regional carbapenem-consumption data [27]. Increased antibiotic pressure may contribute to the selection and dissemination of resistance genes [28]. These factors represent possible explanations for the observed regional patterns but were not directly evaluated in the present study.

By integrating time-series trend analyses, we systematically delineated the evolving characteristics of the AMR epidemiological burden and its potential drivers. Joinpoint analysis demonstrated distinct temporal patterns in the carbapenem-related burden. The ASMR associated with carbapenem resistance increased most rapidly during 2004–2012 (APC = 8.33%; 95% CI: 7.40–10.86%), whereas the fastest increase in the ASMR attributable to carbapenem resistance occurred during 2009–2012 (APC = 10.62%; 95% CI: 4.43–11.83%). Both measures subsequently declined during 2018–2021. Similar declines were observed across most antibiotic classes during the final Joinpoint segments. These recent declines should be interpreted cautiously because they coincided with the COVID-19 pandemic and covered only a short period. Although COVID-19-related infections were excluded from the LRI estimates, pandemic-related changes in healthcare-seeking behaviour, hospital admissions, diagnostic testing, microbiological sampling, surveillance, and mortality reporting may have indirectly affected the estimates of non-COVID-19 infectious disease burden. Because no pandemic-specific adjustment or sensitivity analysis was performed, it remains unclear whether these declines represent true epidemiological improvements, pandemic-related disruptions, or a combination of both.

Although this study provides a comprehensive analysis of the global and regional burden of AMR in *K. pneumoniae*-associated LRI, several limitations should be noted. First, the non-AMR-specific *K. pneumoniae*-associated LRI estimates and the AMR-associated estimates were generated by separate GBD modelling components and are not numerically nested. Consequently, the AMR-associated estimates should not be divided by the non-AMR-specific estimates to calculate the proportion of deaths involving resistance. Differences between these estimates may reflect distinct data inputs, modelling assumptions, and counterfactual definitions. Second, GBD estimates rely on heterogeneous data sources and statistical modelling, particularly in high-burden regions with limited microbiological surveillance. Statistical imputation and changes in diagnostic methods, clinical definitions, healthcare access, and surveillance systems over 1990–2021 may have affected the precision and temporal comparability of the estimates. Third, no separate adjustment or sensitivity analysis was performed to quantify the potential effects of the COVID-19 pandemic on the 2020–2021 estimates. The steep decline observed during the final Joinpoint segment should therefore be interpreted cautiously because it may partly reflect pandemic-related changes in healthcare utilization, surveillance, reporting, or GBD model inputs. Finally, the absence of molecular and genomic data limited our ability to examine specific resistance mechanisms and the dissemination of resistant clones.

## 5. Conclusions

This study characterized the global burden and temporal trends of AMR-related mortality from *K. pneumoniae*-associated LRI between 1990 and 2021. Despite an overall decline in mortality, substantial age-related and geographic disparities persisted, and the burden associated with carbapenem resistance increased in contrast to the declines observed for most other antibiotic classes. Addressing carbapenem resistance therefore remains an important public health priority. Strengthened surveillance in high-burden regions, improved access to rapid diagnostics, appropriate antimicrobial use, and international cooperation are needed to reduce the AMR burden.

## Data Availability

The aggregated and de-identified data analyzed in this study are publicly available through the Institute for Health Metrics and Evaluation (IHME) Global Health Data Exchange and the GBD Results Tool (https://ghdx.healthdata.org/gbd-results-tool, accessed on 10 August 2025). No identifiable individual-level data were accessed or analyzed in this study.

## Supplementary Materials

The following supporting information can be downloaded at https://www.mdpi.com/article/10.3390/tropicalmed11080222/s1, Figure S1: Global temporal trends in ASMR of *Klebsiella pneumoniae*-associated lower respiratory infections across six antibiotic classes, 1990–2021. Bar labels represent all-age ASMR for the six antibiotic classes: (A) associated with bacterial AMR, and (B) attributable to bacterial AMR; Figure S2: Mortality burden of *Klebsiella pneumoniae*-associated lower respiratory infections in 2021, by age group, sex, and GBD super-regions, stratified by six antibiotic classes. Bar labels indicate the number of deaths associated with bacterial AMR; Figure S3: Mortality burden of *Klebsiella pneumoniae*-associated lower respiratory infections in 2021 by age groups, sex, and six antibiotic classes. Bar labels represent count of deaths (A) associated with and (B) attributable to bacterial AMR; Figure S4: Mortality burden of *Klebsiella pneumoniae*-associated lower respiratory infections in 2021, by age group, sex, globally, and GBD super-regions, stratified by six antibiotic classes. Bar labels indicate the number of deaths attributable to bacterial AMR; Figure S5: Joinpoint regression analysis of ASMR trends for *Klebsiella pneumoniae*-associated lower respiratory infections associated with bacterial AMR, globally, stratified by sex, 1990–2021; Figure S6: Joinpoint regression analysis of ASMR trends for *Klebsiella pneumoniae*-associated lower respiratory infections attributable to bacterial AMR, globally, stratified by sex, 1990–2021; Table S1: Burden of *Klebsiella pneumoniae*-associated lower respiratory infections, 1990 and 2021, by GBD region; Table S2: Age-specific mortality trends for *Klebsiella pneumoniae*-associated lower respiratory infections, 1990 to 2021; Table S3: Burden of drug-resistant *Klebsiella pneumoniae*-associated lower respiratory infections, 1990 and 2021, by GBD region; Table S4: Burden of drug-resistant *Klebsiella pneumoniae*-associated lower respiratory infections in 1990 and 2021, stratified by antibiotic class and GBD region; Table S5: Global burden associated with bacterial antimicrobial resistance in *Klebsiella pneumoniae*-associated lower respiratory infections in 1990 and 2021, among children under 5 years and adults over 70 years, by GBD region; Table S6: Global burden attributable to bacterial antimicrobial resistance in *Klebsiella pneumoniae*-associated lower respiratory infections in 1990 and 2021, among children under 5 years and adults over 70 years, by GBD region; Table S7: Global burden associated with bacterial antimicrobial resistance in *Klebsiella pneumoniae*-associated lower respiratory infections in 1990 and 2021, among children under 5 years and adults over 70 years, stratified by antibiotic class and GBD region; Table S8: Global burden attributable to bacterial antimicrobial resistance in *Klebsiella pneumoniae*-associated lower respiratory infections in 1990 and 2021, among children under 5 years and adults over 70 years, stratified by antibiotic class and GBD region; Table S9: Temporal joinpoint analysis of ASMR of *Klebsiella pneumoniae*-associated lower respiratory infections, associated with and attributable to antimicrobial resistance, 1990–2021; Table S10: Temporal joinpoint analysis of ASMR of *Klebsiella pneumoniae*-associated lower respiratory infections, associated with antimicrobial resistance, stratified by antibiotic class, 1990–2021; Table S11: Temporal joinpoint analysis of ASMR of *Klebsiella pneumoniae*-associated lower respiratory infections, attributable to antimicrobial resistance, stratified by antibiotic class, 1990–2021.
